# Impact of disease characteristics and knowledge on public risk perception of zoonoses

**DOI:** 10.1098/rsbl.2022.0148

**Published:** 2022-08-03

**Authors:** Caroline E. Spence, Sarah C. Jenkins, Magda Osman

**Affiliations:** ^1^ Department of Psychology, Queen Mary University of London, London, UK; ^2^ Department of Psychology, Royal Holloway University of London, London, UK; ^3^ Centre for Science and Policy, Judge Business School, University of Cambridge, Cambridge, UK

**Keywords:** zoonoses, risk perception, SARS-CoV-2, attitudes, human–animal interaction, psychometric paradigm

## Abstract

Zoonoses represent a global public health threat. Understanding lay perceptions of risk associated with these diseases can better inform proportionate policy interventions that mitigate their current and future impacts. While individual zoonoses (e.g. bovine spongiform encephalopathy) have received scientific and public attention, we know little about how multiple zoonotic diseases vary relative to each other in lay risk perceptions. To this end, we examined public perceptions of 11 zoonoses across 12 qualitative attributes of risk among the UK public (*n* = 727, volunteer sample), using an online survey. We found that attribute ratings were predominantly explained via two basic dimensions of risk related to public knowledge and dread. We also show that, despite participants reporting low familiarity with most of the diseases presented, zoonoses were perceived as essentially avoidable. These findings imply that infection is viewed as dependent upon actions under personal control which has significant implications for policy development.

## 1. Introduction

Estimates suggest that zoonoses, pathogens transferred between animals and humans, cause around 2.5 billion cases of illness and 2.7 million deaths globally per year [1]. Given that 75% of emerging infectious diseases are zoonotic in origin [2], meaningful reductions in the magnitude of zoonoses-associated health threats appears unlikely in the near-future, with SARS-CoV-2 having caused approximately two million deaths in 2020 alone [3]. Beyond these substantial risks to human morbidity and mortality, zoonotic diseases generate indirect losses in affected economies estimated at US $200 billion [4]. The time, money and effort expended by governments in assessing and reacting to zoonoses is therefore of consequence. In parallel to the assessments generated by governmental bodies/experts evaluating risks to public health and global security, non-expert perceptions of zoonoses are also known to have repercussions for humans and animals as part of society's response to zoonotic hazards and their impacts [5]. For example, public fear during the UK bovine spongiform encephalopathy (BSE) outbreak in the 1980s contributed to the slaughter of 3.3 million cattle [6], extensive surveillance programmes and a ban on British beef exports entering Europe; despite expert opinion having voiced concerns that this reaction was disproportionate compared to that seen for other zoonoses [7]. Therefore, people's perceptions and subsequent behaviour likely play a pivotal role not only in direct exposure, transmission and control of zoonotic diseases [8,9] but also in mitigating their consequences more broadly across society for both humans and animals. Previous research seeking to aid policy formulation in this area has typically concentrated on public attitudes and understanding of the potential risks of one or two specific zoonoses at a time [10,11]. Yet, we know little of the manner in which lay perceptions of risk vary across differing zoonotic diseases, a likely important factor when considering where to target finite resources. We also know that when fictional zoonoses with contrasting characteristics (e.g. pathogen type, symptoms) are presented simultaneously, lay perceptions of (i) perceived risk, and (ii) appropriate disease management strategies differ [12]. Notably, this single-hazard approach regarding zoonoses contrasts with other areas of research on risk perception where a comparative approach, in which individuals evaluate risks across numerous hazards, is often evident [13]. Indeed, research investigating risk perceptions commonly employs the psychometric paradigm, developed by Fischoff *et al*. [14,15], to produce ‘cognitive maps’ of multiple hazards via lay assessments of subjective risk attributes such as ‘newness’ and ‘voluntariness’. This body of work has identified two key components, each combining multiple qualitative attributes, that underlie lay perceptions of risk. Termed ‘dread’ and ‘unknown’, the former comprises attributes such as ‘fear’ and ‘voluntariness’, while the latter is associated with ‘newness’ and unknown or delayed impacts. This two-dimensional characterization of lay risk perception has held across topics ranging from food hazards [16] to pharmaceuticals [17], as well as across cultures [18] and time [19]. The psychometric paradigm was used in the present exploratory study to investigate perceptions of risk across zoonoses among the public; namely do zoonotic risk perceptions conform to the previously documented dimensions of ‘dread’ and ‘unknown’? Additionally, given their reported impact in previous work comparing fictional zoonoses, we also aimed to assess how variation in the characteristics of real zoonoses might alter people's judgements of associated risk.

## 2. Material and methods

### (a) Participants

Participants were recruited using the crowdsourcing website Prolific [20] and were required to have been born in the UK.^1^ Participation was via self-selection and participants were compensated £6.50 per hour. After exclusions (see electronic supplementary material), the final sample comprised 727 participants (444 women, 274 men, nine other; modal age group: 25–34 years). Sample characteristics, extended methods and extended data analyses are outlined in the electronic supplementary material.

### (b) Questionnaire and procedure

Zoonoses were selected for investigation according to two criteria: firstly, government data [22] reports the disease as occurring in the UK, and secondly, the zoonoses were required to show variation across a number of specified characteristics, namely host animal, pathogen type and route of infection, in order to investigate their potential influence on associated judgements of risk (table 1). Where a single zoonosis was associated with multiple host species or routes of infection, the animal/route likely causing the most infections among the UK public was chosen. On the basis that participants might have little pre-existing knowledge of some zoonoses [23], each zoonoses was presented with some brief facts relating to the specified characteristics (see electronic supplementary material), though no information regarding risk was provided.

**Table 1.** Zoonoses characteristics.

zoonoses (abbreviation)	common name	pathogen	host species	route of infection
leptospirosis (LEP)	Weil's disease	bacteria	rats	direct or indirect contact with urine of infected animals
pasteurellosis (PAS)	—	bacteria	dogs and cats	bites and/or scratches
psittacosis (PSI)	parrot fever/ornithosis	bacteria	birds	inhalation of dust particles from dried faeces or feathers
borreliosis (BOR)	Lyme disease	bacteria	ticks that live on mammals and birds	tick bite
lyssavirus (LYS)	bat rabies	virus	bats	bites and/or scratches
hepatitis E (HEP)	HEV	virus	pigs	consumption of contaminated food or accidental ingestion of faecal material from infected animals (faecal–oral route)
SARS-CoV-2 (COV)	COVID-19	virus	unknown	inhalation of respiratory droplets from infected animals
variant Creutzfeldt–Jakob disease (CJD)	vCJD	prion	cows	consumption of meat from cows with BSE
dermatophytosis (DER)	ringworm	fungus	range of animals	direct contact with infected animals and/or surfaces contaminated by those animals
echinococcosis (ECH)	hydatid disease	parasite: tapeworm	dogs	accidental ingestion of faecal material from infected animals (faecal–oral route)
toxoplasmosis (TOX)	—	parasite: single celled	cats	accidental ingestion of faecal material from infected animals (faecal–oral route)

To investigate whether risk perceptions across multiple zoonoses conform to the predicted dimensions of ‘dread’ and ‘unknown’, zoonoses were rated on a total of 11 risk attributes (table 2), using seven-point Likert-style response scales. Attributes were selected on the basis of common use in the psychometric literature [10,15,19,24–30] and their relevance to zoonotic diseases. Participants were also asked to provide an overall risk rating for each zoonosis, e.g. ‘Toxoplasmosis is …’ rated on a seven-point scale from ‘not at all risky’ (1) to ‘highly risky’ (7) (as in MacDaniels *et al*. [31]). Given that risk perceptions may vary dependent on risk target (e.g. individual versus population) [32], participants were required to consider how great a risk ‘to the general population of the UK’ each zoonosis was. Presentation of both individual zoonoses and attribute response scales were randomized as was the presentation order of response scales for ‘overall risk’ (either before or after the attribute response scales for each zoonoses). The questionnaire was hosted and administered online via Qualtrics [33].

**Table 2.** Risk rating attributes. Text in parentheses represents anchor points of the response scale (1–7). Attributes are illustrated using the example of toxoplasmosis.

All scale items commenced 'To what extent…'
**Voluntary**
Do people take on the risks associated with contracting toxoplasmosis voluntarily?
(Completely voluntary–completely involuntary)
**Known to those exposed**
Are the risks associated with toxoplasmosis known by those who are exposed to it?
(Known precisely–not known at all)
**Known to science**
Are the risks associated with toxoplasmosis known to science?
(Known precisely–not known at all)
**Familiarity**
Are you familiar with the health risks associated with contracting toxoplasmosis?
(Very familiar–totally unfamiliar)
**Response efficacy**
Can people take effective actions to avoid contracting toxoplasmosis?
(Very much–not at all)
**Naturalness**
Are the risks associated with toxoplasmosis natural, or the fault of mankind?
(Natural–man is to blame)
**Newness**
Are the risks associated with toxoplasmosis old risks or new risks?
(Very old–very new)
**Likelihood of harm to health**
Is toxoplasmosis likely to harm the health of those who contract it?
(Very mild harm–very serious harm)
**Fear**
Is toxoplasmosis a risk that is strongly feared?
(No fear at all–strong fear)
**Institutional trust**
Do public health authorities in the UK have the capacity to deal with an outbreak of toxoplasmosis?
(Very high capacity–very low capacity)
**Regulation**
Does toxoplasmosis need to be controlled by regulatory measures?
(No regulation needed–strict/extensive regulation needed)

## 3. Results

### (a) Principal component analysis

With the aim of replicating analysis methods previously reported within the psychometric paradigm, e.g. [14,34,35], principal component analysis (PCA) was used to explore the data structure (as opposed to exploratory factor analysis; see Extended analyses in electronic supplementary material, for further discussion). Analyses were performed at the aggregate level, with data collapsed across individual zoonoses [36] in order to address the central question of do zoonotic risk perceptions conform to the previously documented dimensions of ‘dread’ and ‘unknown’? The resulting data matrix was used to generate a correlation matrix for the 11 risk attributes to be included in the PCA. All attributes showed at least one correlation above 0.3 with the exception of ‘voluntary’, which was subsequently excluded from further analysis, as recommended by Field [37]. PCA on the remaining 10 attributes using a Varimax rotation revealed a three-component solution, which explained 58.2% of the total variance. The component loading matrix is presented in table 3.

**Table 3.** PCA loadings and Cronbach's alpha (*α*) of risk attributes.

attribute	component		
	1 societal knowledge	2 dread	3 personal knowledge
known - science	**0.822**		
newness	**0.815**		
naturalness	**0.591**		
response efficacy	**0.552**		
institutional trust	**0.489**		
likelihood harm		**0.828**	
regulation		**0.748**	
fear		**0.660**	−0.414^a^
known - exposed			**0.853**
familiarity			**0.673**
Cronbach's alpha	0.682	0.659	0.544^a^

^a^Owing to cross-loading, ‘fear’ was assigned to the component with the highest loading (component 2) resulting in a two-item scale for component 3. As a result, the Spearman–Brown split-half reliability coefficient for the component was calculated alongside Cronbach's coefficient alpha as recommend by Eisinga *et al*. [38] (SB coefficient = 0.545). Deletion of items did not increase Cronbach's alpha for components 1 and 2 (n.a. for component 3). *Note*: Only loadings above 0.4 were interpreted [39].

**Figure 1.**
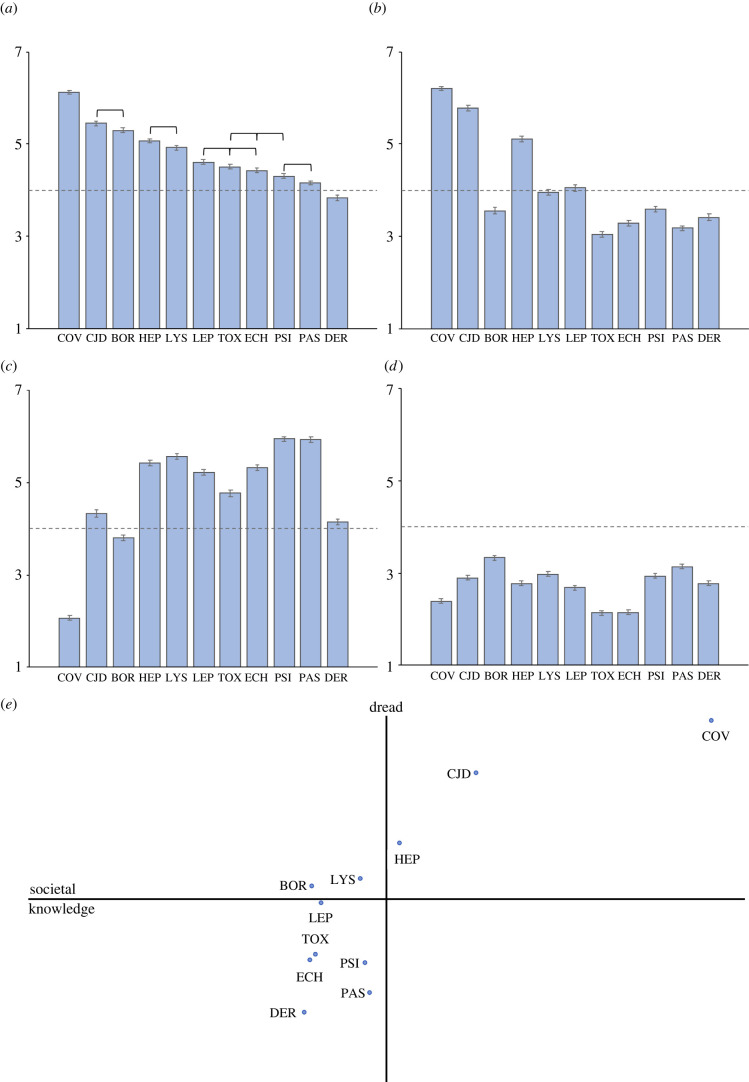
Attribute ratings and location of zoonoses within component space. Mean rating of each zoonoses for (*a*) ‘overall risk’, (*b*) ‘regulation’, (*c*) ‘familiarity’ and (*d*) ‘response efficacy’. *Y*-axis represents seven-point response scale. Dotted line represents scale midpoint. Error bars represent s.e.m. Figure (*a*) only: connecting lines indicate non-significant (greater than 0.01) post-hoc analysis result—all other pairwise comparisons were significant. *Note*: connecting lines exclude SARS-CoV-2 and dermatophytosis which were significantly different from all other zoonoses. (*e*) Location of zoonoses within two-component space. BOR, borreliosis; COV, SARS-CoV-2; CJD, variant Creutzfeldt–Jakob disease; DER, dermatophytosis; ECH, echinococcosis; HEP, hepatitis E; LEP, leptospirosis; LYS, lyssavirus; PAS, pasteurellosis; PSI, psittacosis; TOX, toxoplasmosis.

The first component, labelled ‘societal knowledge’, contained the attributes ‘known to science’, ‘newness’, ‘naturalness’, ‘response efficacy’ and ‘institutional trust’ (Cronbach's *α* = 0.682, variance explained = 23.6%). The second component, labelled ‘dread’, contained the attributes of ‘likelihood of harm’, ‘regulation’ and ‘fear’ (*α* = 0.659, variance explained = 18.4%). The final component was termed ‘personal knowledge’, with the ‘known to those exposed’ and ‘familiarity’ attributes loading onto this component (*α* = 0.544, variance explained = 16.2%). All components produced an alpha above the 0.5 recommended by Nunnally [40] for exploratory research. Component scores for each zoonosis were calculated, with the component space of the first two components being shown in figure 1*e*. On the basis that (i) the origin of SARS-CoV-2 is unconfirmed, (ii) transmission is primarily human–human, and (iii) current salience might generate excessive influence, we repeated the PCA excluding SARS-CoV-2 data (electronic supplementary material, table S2). The results indicated no change to the three-component structure. Owing to the predominance of female participants, analyses were also re-run according to sex, which again did not change the three-component structure (electronic supplementary material, tables S3–S4).

### (b) Risk ratings

Turning to our second objective of investigating judgements of risk across zoonoses and the role that variation in zoonoses characteristics (i.e. pathogen type, host animal and route of infection) might play; SARS-CoV-2, vCJD and borreliosis received the highest mean ‘overall risk’ ratings (*M* ± s.d. = 6.12 ± 1.16, 5.45 ± 1.38, and 5.30 ± 1.20, respectively) while dermatophytosis was considered to be the least risky zoonosis (3.83 ± 1.48, figure 1*a*). ‘Overall risk’ ratings were found to be significantly different between zoonoses, Welch's *F*_10, 3160.181_ = 194.824, *p* < 0.0001, *ω*^2^ = 0.18. Games–Howell post-hoc analysis revealed that SARS-CoV-2 was rated as significantly more risky and dermatophytosis as significantly less risky than all other zoonoses (*p*-values < 0.001). All other pairwise comparisons are summarized in figure 1*a* (see electronic supplementary material, table S6 for full results of all analyses). Overall risk ratings were also found to differ significantly according to pathogen type, Welch's *F*_4, 1780.626_, *p* < 0.0001, *ω*^2^ = 0.21, with zoonoses caused by prions or viruses perceived as significantly riskier compared to those from bacteria, parasites or fungi (*p-*values < 0.0001, see electronic supplementary material, table S7).

Multiple regression analyses predicting the ‘overall risk’ of each zoonosis from the 10 attribute ratings indicated that ‘likelihood of harm’, ‘fear’ and ‘regulation’ were significant predictors across all zoonoses (*p-*values < 0.01, electronic supplementary material, tables S8–S18). However, mean ratings on the perceived need for regulation attributed to each zoonosis did not mirror associated mean ‘overall risk’ ratings; for example, while borreliosis was rated the third highest for ‘overall risk’, it received the seventh highest rating for ‘regulation’ (figure 1*a,* ‘overall risk’ versus figure 1*b,* ‘regulation’). The zoonoses considered least in need of regulation were all listed as transmitted by pets (toxoplasmosis, pasteurellosis and echinococcosis). By contrast, of the three zoonoses considered most in need of regulation, two were listed as transmitted by farm animals (vCJD and hepatitis E).

Mean attribute ratings for individual zoonoses indicated that participants identified as unfamiliar with the health risks associated with all zoonoses except SARS-CoV-2 and borreliosis (figure 1*c,* ‘familiarity’). Furthermore, participant ratings for the ‘response efficacy’ attribute failed to reach the midpoint for all zoonoses (figure 1*d,* ‘response efficacy’), indicating the widespread belief that people can take effective action to prevent all zoonotic infections. With the exception of SARS-CoV-2, all zoonoses that were considered the most preventable (i.e. received low ‘response efficacy’ ratings) were listed as transmitted by contact with excreta (toxoplasmosis, echinococcosis, leptospirosis and hepatitis E).

## 4. Discussion

Zoonoses are widespread, increasing in prevalence, and have significant health and economic impacts [41]. As an accepted precursor to risk mitigation behaviour [42], accurate understanding of public risk perception is therefore important. However, knowledge of risk perception across differing zoonoses remains limited, hindering proportionate decisions regarding resource targeting. To support progress, the present study explored public perceptions of risk alongside the influence of disease characteristics on judgements across 11 zoonoses using the psychometric paradigm.

Along with the components ‘unknown’ and ‘dread’, consistent with Fischoff and colleagues' original work, the present study also revealed a third component ('personal knowledge'). Personal knowledge subsumes the attributes ‘familiarity’ and ‘known to those exposed’. Thus, we relabelled the original ‘unknown’ component as ‘societal knowledge’, distinguished from the new component ‘personal knowledge’. Recall, the sample was asked to provide risk ratings according to ‘risk to the general population of the UK’ (societal-level risk, third-person perspective). However, the attribute ‘familiarity’ asked ‘are you familiar with the health risks' (individual-level risk, first-person perspective) for specified zoonoses. Additionally, given the high level of human–animal interaction in UK (e.g. pet ownership [43], widespread meat consumption [44]) and exposure of all participants to at least one of the zoonoses presented (SARS-CoV-2), we propose that the attribute statement ‘known to those exposed’ was also interpreted from a first-person perspective. Essentially, these two risk ratings likely prompted respondents to draw upon knowledge of a personalized nature, splitting the originally reported ‘unknown’ component to produce the third component. This is in line with the impersonal impact hypothesis [45,46], which suggests that judgements of risk at a societal versus personal level are largely distinct, with mass media predominantly influencing societal-level judgements while individual judgements focus on personal experience.

Participants were largely unfamiliar with the health risks associated with zoonoses, with the exception of SARS-CoV-2 and borreliosis. This lack of familiarity potentially hinders realistic risk estimates among the public, generating a reliance on heuristics in the absence of relevant knowledge. For instance, for all zoonoses, the ‘dread’ component (combining the ‘likelihood of harm to health’, ‘fear’ and ‘regulation’ attributes) was found to underpin ‘overall risk’ ratings. Zoonoses transmitted by dogs and cats were also perceived as least in need of regulation, despite frequent contact between pets and the public. These findings corroborate public use of heuristics related to affect [47] in judgements of perceived risk. Given the significant differences found in mean overall risk ratings between zoonoses, which (based on participants' lack of familiarity) we assume are a product of the characteristics information available during the study, awareness of the types of information likely to generate these heuristic-based assessments is essential in future zoonoses policy communication [48].

Unexpectedly, despite reporting low familiarity, participants strongly believed individuals could take effective action to avoid contracting all zoonotic infections (response efficacy) with ratings failing to reach the scale midpoint for any zoonosis. Potentially, the nature of information provided on disease characteristics (i.e. route of transmission) could have prompted judgements that all contact with animals, their meat or excreta, and therefore infection, is optional and avoidable. However, people's predisposition to make internal/person-focused attributions rather than situational/external ones [49] and ‘optimism bias’ [50] likely explain why zoonotic infection is attributed to action taken by individuals. This finding aligns with the increased attribution of responsibility seen to those infected with SARS-CoV-2 [51], and suggests that ‘victim-blaming’ also occurs for other zoonoses.

Despite attempts to account for sample limitations in our analyses, it is important to acknowledge our findings are based on a non-probability sample. In delineating a specific population for initial, exploratory research, it is not possible to claim the findings are representative of all individuals in the UK. Nevertheless, our study provides a platform for broader exploration of zoonotic risk perception in relation to disease characteristics, demographics variations and potential victim-blaming. The implications here are that, given the lack of public knowledge regarding zoonoses, effective communication strategies must account for widespread use of heuristics in relation to associated judgements of risk so as to avoid potential victim-blaming and misattribution of agency and control.

## Data Availability

Data are available from the Dryad Digital Repository at https://doi.org/10.5061/dryad.dncjsxm32 [52].

## Endnote

1 Representing the majority (86%) of the UK population [21].
